# “Nature Abhors a Vaccuum”: Invagination of the Small Intestine into the Lumbar Disc Space After a Spinal Fusion Operation

**DOI:** 10.3390/diagnostics10040185

**Published:** 2020-03-27

**Authors:** Wonho Lee, Mathieu Boudier-Revéret, Du Hwan Kim, Min Cheol Chang

**Affiliations:** 1 Department of Radiology, Topspine Hospital, Daegu 41931, Korea; yiseul1@gmail.com; 2 Department of Physical Medicine and Rehabilitation, Centre hospitalier de l’Université de Montréal, Montreal, H2W 1T8, Canada; 3 Department of Physical Medicine and Rehabilitation, College of Medicine, Chung-Ang University, Seoul 06973, Korea; ri-pheonix@hanmail.net; 4 Department of Physical Medicine and Rehabilitation, College of Medicine, Yeungnam University, Daegu 42415, Korea

**Keywords:** invagination, small intestine, spinal fusion, spinal instability, intervertebral disc

## Abstract

A 77-year-old woman having back pain due to an L2 vertebral body compression fracture took a lumbar spine magnetic resonance imaging (MRI). In MRI, in addition to the L2 vertebral body fracture, invagination of the small intestine into the intervertebral disc space at L5-S1 was found by chance. On a lateral lumbar spinal X-ray, the lordotic angle was markedly increased at the L5-S1 level. Additionally, the L5-S1 disc space had widened. These X-ray findings indicate the segmental instability at L5-S1. The spinal fusion operation on L3-4-5 seems to have resulted in overt mechanical loading on the inferior spinal segment (L5-S1). We think the instability damaged the anterior longitudinal ligament and caused a tear in the anterior portion of the annulus fibrosus. The defect in the L5-S1 intervertebral disc after the tear would have caused the vacuum, which is presumed to have pulled the patient’s small intestine into the empty space within the L5-S1 intervertebral disc. Although intervertebral invagination of intra-abdominal structures is not common, clinicians should be aware of the possibility of this complication in patients who have spinal segmental instability.

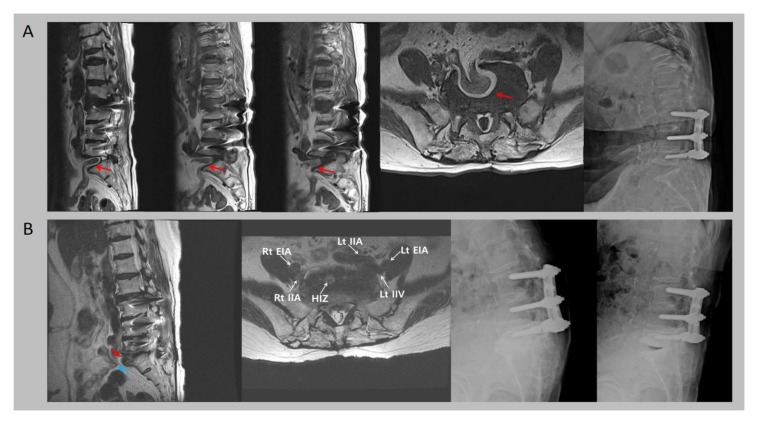



**Figure 1.** (**A**) T2-weighted lumbar spine magnetic resonance images (MRIs) on admission day reveal invagination of the small intestine (red arrows) into the intervertebral disc space of L5-S1. A lateral lumbar spinal X-ray performed on the day of admission shows the markedly increased lordotic angle at the L5-S1 level and a widened L5-S1 disc space. (**B**) T2-weighted MRI from 8 years ago reveals degeneration and a high-intensity zone (red arrow) on the L5-S1 intervertebral disc, and flexion and an extension lateral X-ray showed spinal segmental instability at L5-S1. Blue arrow: small bowel loop. External iliac artery (EIA); internal iliac artery (IIA); high-intensity zone (HIZ). A 77-year-old woman was admitted to a local hospital complaining of back pain resulting from a L2 acute vertebral body compression fracture after falling 1 month previously. She had received a posterior lumbar interbody fusion at the L3-4-5 levels due to spinal stenosis 20 years before. On the day of admission, a lumbar spine magnetic resonance imaging (MRI) showed that in addition to the L2 vertebral body fracture, invagination of the small intestine into the intervertebral disc space at L5-S1 was discovered by chance (Figure 1A). However, the symptom of a bowel obstruction was not observed. On a lateral lumbar spinal X-ray, the lordotic angle was markedly increased at the L5-S1 level (Figure 1A). Additionally, the L5-S1 disc space had widened. On retrospective review, on an MRI taken 8 years previously, the high-intensity zone was observed at the anterior portion of the L5-S1 intervertebral disc (Figure 1B). In addition, a flexion and extension lateral lumbar spinal X-ray performed 8 years ago showed segmental instability at L5-S1 and an increased lordotic angle, respectively, though the degree of lordosis was less pronounced (Figure 1B). Over the following 8 years, the segmental instability at L5-S1 had aggravated. Additionally, the X-rays taken on admission 8 years before revealed a broken L5 screw, which suggests that the L5 screw had not been functioning. The T-score of bone mineral density of the L1-4 spine on admission was −5.6, indicating severe osteoporosis. The invagination of peritoneal or retroperitoneal structures into the lumbar intervertebral disc space rarely occurs. A dozen cases of intervertebral invagination of intra-abdominal structures, such as the intestine, vena cava, iliac vessels, the torn redundant anterior longitudinal ligament (ALL), retroperitoneal fat, and the psoas muscle have been reported [1,2,3,4]. Usually, invagination of peritoneal or retroperitoneal structures into the lumbar intervertebral disc space are induced by acute trauma. Only two studies have reported atraumatic cases of entrapment of intra-abdominal structures into the intervertebral disc space [2,4]. A defect or severe laxity of the anterior longitudinal ligament (ALL) combined with disruption of the anterior portion of the annulus fibrosus are prerequisites for the invagination of intra-abdominal structures into the intervertebral disc space [2]. Even though these anterior vertebral structures contribute to spine stability, enabling it to withstand high mechanical loads, continuous mechanical forces due to spinal segmental instability can damage the ALL and the anterior annulus fibrosus. Spinal fusion is a frequently performed surgical technique to treat various spinal conditions. However, after surgery, rapid degeneration of the adjacent spinal segments can occur. This can lead to various spinal disorders, including spinal stenosis, herniated lumbar disc, and hypertrophic facet arthritis [5]. Likewise, for our patient, the spinal fusion operation on L3-4-5 resulted in overt mechanical loading on the inferior spinal segment (L5-S1) with associated segmental instability. The instability may have contributed to damaging the ALL and caused a tear in the anterior portion of the annulus fibrosus (as we can see in the MRI from 8 years ago). The defect in the L5-S1 intervertebral disc after the tear would have caused the vacuum, which was filled with the patient’s small intestine. Although intervertebral invagination of intra-abdominal structures does not commonly occur, clinicians should be aware of this complication in patients who have spinal segmental instability, especially in those who have undergone spinal fusion surgery.
